# Idiopathic Segmental Megacolon—Intestinal Neuronal Dysplasia Type B, Cajal Cells Abnormality, or Still “Idiopathic” Disease?—Case Report and Literature Review

**DOI:** 10.3390/reports9040339

**Published:** 2026-10-08

**Authors:** Georgi Popivanov, Boryana Ilcheva, Marina Konaktchieva, Roberto Cirocchi, Mihail Tabakov, Vladimir Pavlov, Ventsislav Mutafchiyski

**Affiliations:** 1 Department of Surgery, Military Medical Academy, 1606 Sofia, Bulgaria; 2 Department of Pathology, Military Medical Academy, 1606 Sofia, Bulgaria; 3 Department of Gastroenterology and Hepatology, Military Medical Academy, 1606 Sofia, Bulgaria; 4 Department of Medicine and Surgery, University of Perugia, 06123 Perugia, Italy; 5 Department of Surgery, UHAT “Sv. Ivan Rilski”, 1000 Sofia, Bulgaria; 6 Department of Surgery, Helios Klinikum Krefeld, 47805 Krefeld, Germany

**Keywords:** idiopathic megacolon, intestinal neuronal dysplasia, Cajal cell abnormalities, case report

## Abstract

**Background and Clinical Significance:** Idiopathic megacolon (IM) in adults is a rare disease with an unknown aetiology. The condition remains poorly understood, with ongoing debate over the specificity of its histopathology. **Case Presentation:** Case 1: A 40-year-old man was admitted due to moderate lower abdominal pain and a distended, asymmetric abdomen. X-ray demonstrated the sigmoid colon with a diameter of 13 cm. Colonoscopy found a significantly enlarged and elongated sigmoid colon, which precluded the examination of the entire colon due to the insufficient length of the colonoscope. All blood assays were within the normal range. Intraoperatively, there was a significantly dilated sigmoid colon measuring 20 cm in width with a length of 60 cm. A sigmoid resection with mechanical T-T anastomosis between the descending colon and proximal rectum circular stapler was performed. The patient had an uneventful recovery. Seven years after the operation, he is symptom-free. The histopathological examination revealed a slight oedema of the lamina propria, submucosal fibrosis, hyperplastic submucosal ganglia and nerve fibres, hypertrophy of the muscle layers and hyperplastic myenteric nerve plexuses and ganglion cells. Case 2: A 28-year-old otherwise healthy man was admitted due to intermittent diffuse abdominal pain that manifested suddenly during a military mission. There was no remarkable medical history. The abdominal contrast CT revealed a segmental sigmoid megacolon with a diameter of 10 cm without any other noteworthy pathology. All organic causes for megacolon were excluded. No conservative treatment was applied. The intraoperative finding was a dilated sigmoid measuring 15 cm in diameter. A sigmoid resection was performed, followed by T-T anastomosis between the descending colon and proximal rectum. During the postoperative course, there was significant anastomotic bleeding, which was managed successfully by blood transfusion and haemostatic clips. The patient was discharged on the 8th postoperative day. Eighteen months after the operation, he is symptom-free. The histopathological examination was performed using Formalin-Fixed Paraffin-Embedded (FFPE) tissue, cut at 3 µm. Immunohistochemistry was performed on an autostainer Leica Bond with ready-to-use reagents—CD117 clone EP10 and S100 clone EP32. The dilated part revealed a fibrotic submucosa with hyperplastic vessels and nonspecific inflammatory changes, hypertrophy of the muscle layers, and hyperplastic submucosal and myenteric ganglia and plexuses, but did not fulfil the criteria for IND. The same picture was observed in both the distal narrowed part and the resection lines. There was no obvious abnormality of the Cajal cells in both parts of the specimen. **Conclusions:** We were unable to identify a specific cause of IM in the presented cases. The histopathological findings were not compatible with IND, Hirschsprung disease, or other neuromuscular pathology. The critical analysis of the literature does not support IND as a possible cause of IM. Due to the heterogeneity of colonic motility disorders and the lack of a consistent histological pattern in IM, we hypothesise that it has a multifactorial aetiology.

## 1. Introduction and Clinical Significance

Idiopathic megacolon (IM) in adults is a rare disease with an unknown aetiology. The most essential prerequisite for diagnosis is the absence of organic causes such as intoxication, hypo- or aganglionosis, trauma, infection, distal stenosis, neurosurgical interventions, etc. It is sporadic, but familial affection has also been reported (five generations with six of 49 members had megacolon in adulthood) [1,2]. The clinical manifestation of IM is nonspecific [1]. The most frequent complaints are constipation (68%), abdominal pain (57%), a feeling of distension (40%), and a decreased quality of life [3,4]. To some extent, the symptoms overlap with the constipation type of irritable bowel syndrome, which hampers timely diagnosis. On the other hand, the differential diagnosis is important because of the different treatment of both conditions. Complications are rare and could include volvulus, faecal impaction, and colonic obstruction [2]. The condition remains poorly understood, with ongoing debate over the specificity of its histopathology.

Herein, we report two well-documented cases of segmental left megacolon and discuss the histopathological findings in the light of the London Classification of gastrointestinal neuromuscular pathology [5].

## 2. Case Presentation

### 2.1. Case 1

A 40-year-old man was admitted due to moderate lower abdominal pain and a distended, asymmetric abdomen. There was no history of past disease, comorbidity, drug use, a recent major trauma or major surgery, mental illness, endocrine disorders, IBD, pseudo-obstruction or infections. X-ray demonstrated a dilated sigmoid colon measuring 13 cm. All blood assays were within the normal range. Colonoscopy found a significantly enlarged and elongated sigmoid colon, which precluded the examination of the entire colon due to insufficient length of the colonoscope. No distal obstruction was found. This finding was confirmed on the subsequent barium enema examination. Thus, all organic causes for megacolon were excluded.

**Figure 1:**
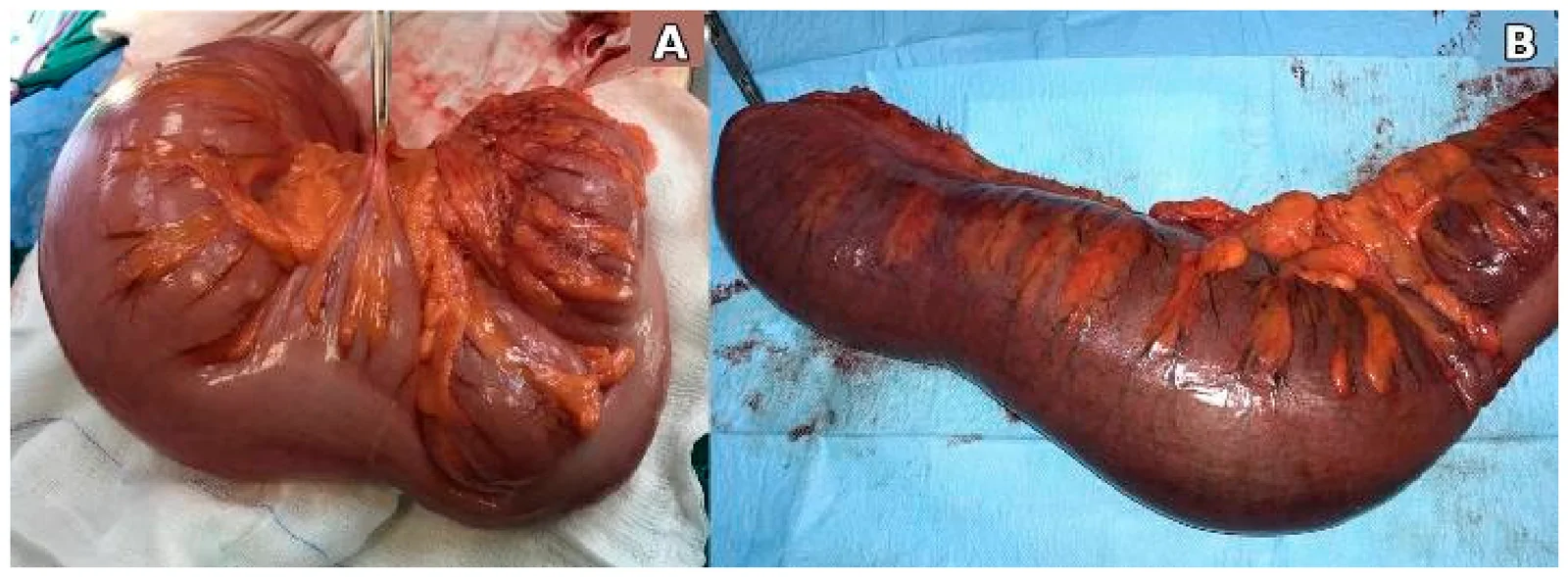
An intraoperative view of the dilated sigmoid colon (**A**) and the resected specimen—dilated sigmoid colon measuring 20 cm in width with a length of 60 cm (**B**).

No conservative treatment was applied. Intraoperatively, there was a significantly dilated sigmoid colon measuring 20 cm in width with a length of 60 cm (Figure 1). A sigmoid resection with mechanical T-T anastomosis between the descending colon and proximal rectum via a 31 mm (4.5 mm) circular stapler was performed. The patient had an uneventful recovery. Seven years after the operation, he is symptom-free.

**Figure 2:**
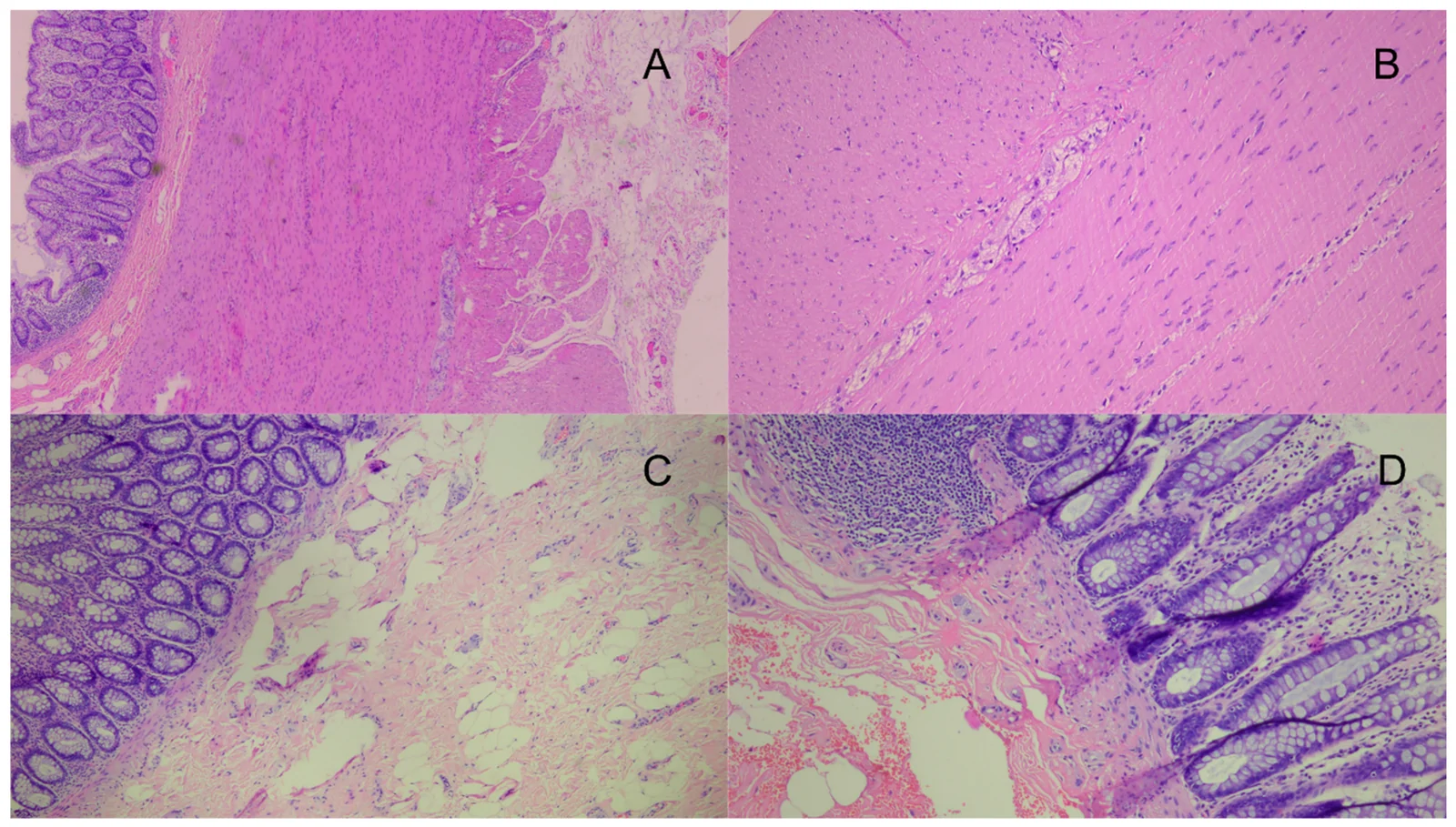
(**A**,**B**) Hyperplastic myenteric nerve plexuses and ganglion cells—haematoxylin–eosin—5× (**A**) and 10× (**B**), (**C**,**D**) hyperplastic submucosal ganglia and nerve fibres—5× (**C**) and 10× (**D**).

The histopathological examination revealed a slight oedema of the lamina propria, fibrosis of the submucosa, hyperplastic submucosal ganglia and nerve fibres, hypertrophy of the muscle layers and hyperplastic myenteric nerve plexuses and ganglion cells (Figure 2). The same finding was observed in the resection margins.

A final diagnosis of idiopathic megacolon was made based on the colonoscopy, intraoperative findings, and histopathology showed no detectable cause for the condition.

### 2.2. Case 2

**Figure 3:**
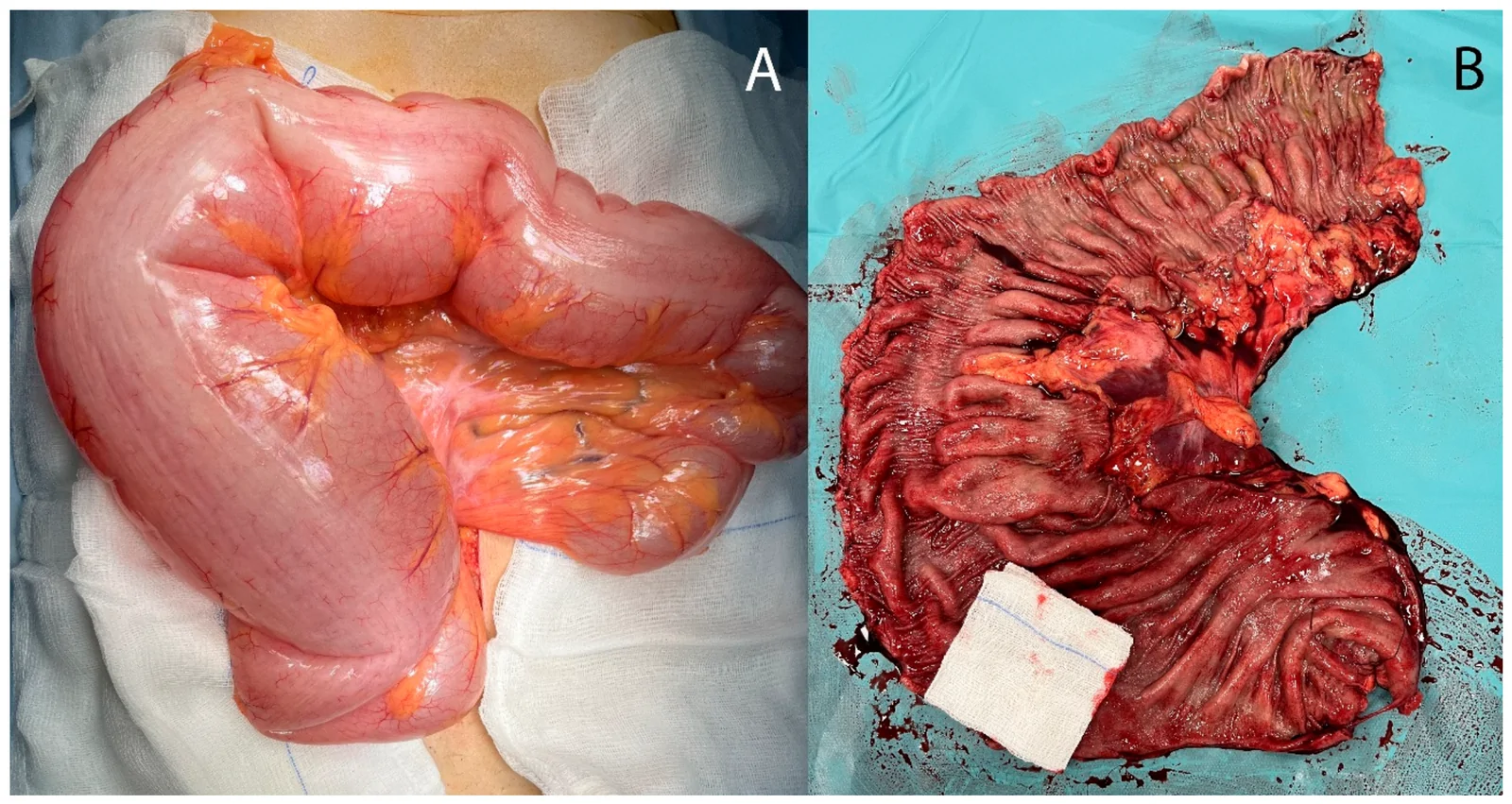
(**A**) An intraoperative view and (**B**) the resected specimen of the sigmoid colon with a maximal diameter of 15 cm.

A 28-year-old military serviceman was admitted due to intermittent diffuse abdominal pain that manifested suddenly during a military mission. He was transferred by air from Spain, reporting two similar previous episodes. There was no history of past disease, comorbidity, drug use, mental illness, endocrine disorders, IBD, pseudo-obstruction (no trauma or major surgery) or infections. All blood assays were within the normal range. The plain X-ray demonstrated sigmoid dilatation to 10 cm. The contrast CT revealed a segmental sigmoid megacolon with fluid-air levels, with a diameter of 10 cm, without any other noteworthy pathology. No conservative treatment was applied. The intraoperative finding was a dilated sigmoid with a maximal diameter of 15 cm (Figure 3). A sigmoid resection was performed, followed by T-T anastomosis between the descending colon and proximal rectum via a 31 mm (4.5 mm) circular stapler. During the postoperative course, there was significant anastomotic bleeding, which was managed successfully by blood transfusion and haemostatic clips (Clavien-Dindo 3b). The patient was discharged on the 8th postoperative day. Eighteen months after the operation, he is symptom-free.

**Figure 4:**
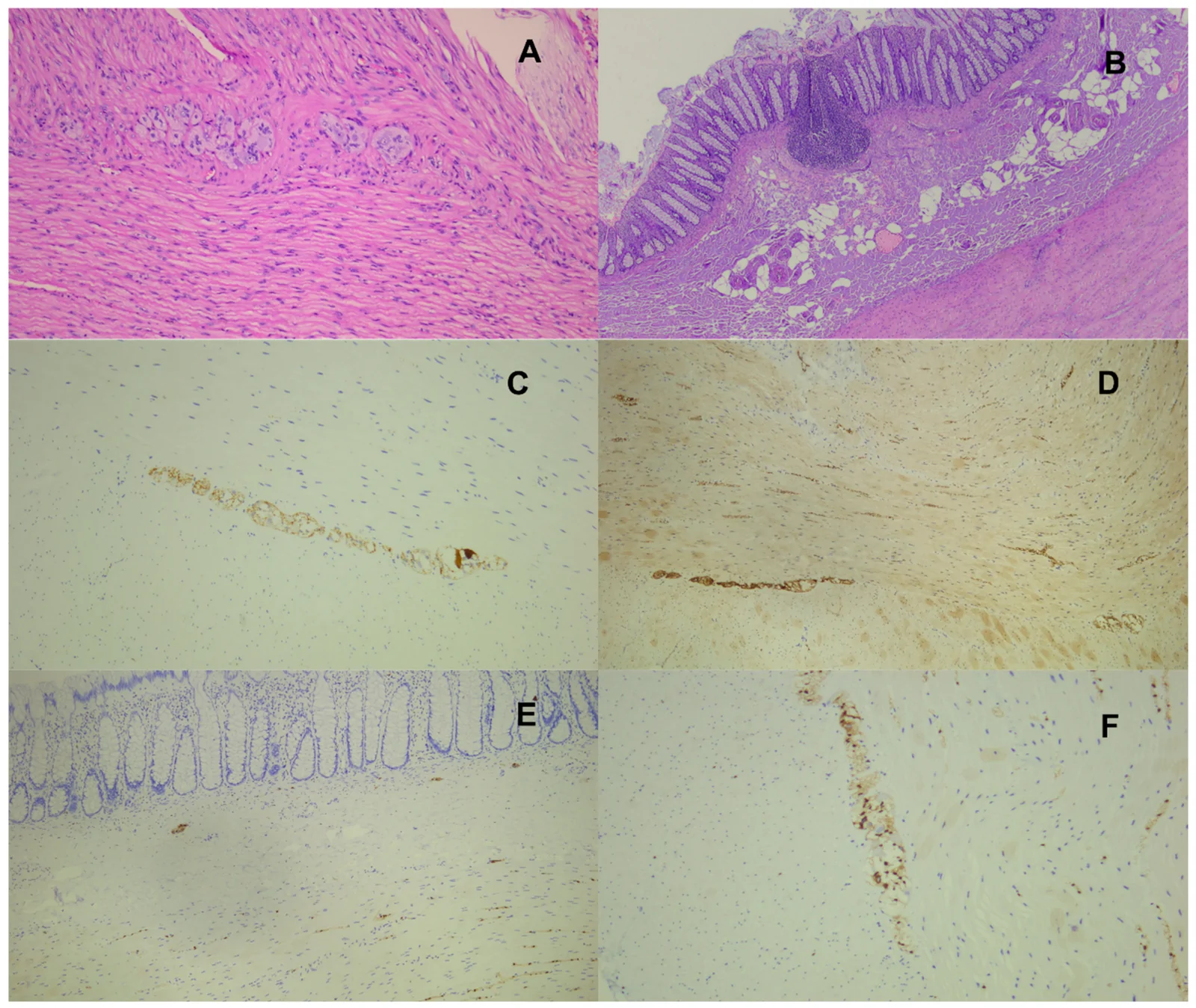
(**A**) hypertrophic myenteric ganglia (HE, 10×), (**B**) hyalinization of the submucosa (HE, 2.5×), (**C**) hypertrophic myenteric ganglia (Calretinin, 5×), (**D**) hypertrophic myenteric ganglia (D2-40, 5×), and (**E**,**F**) hypertrophic submucous and myenteric ganglia (SOX10, 5×).

**Figure 5:**
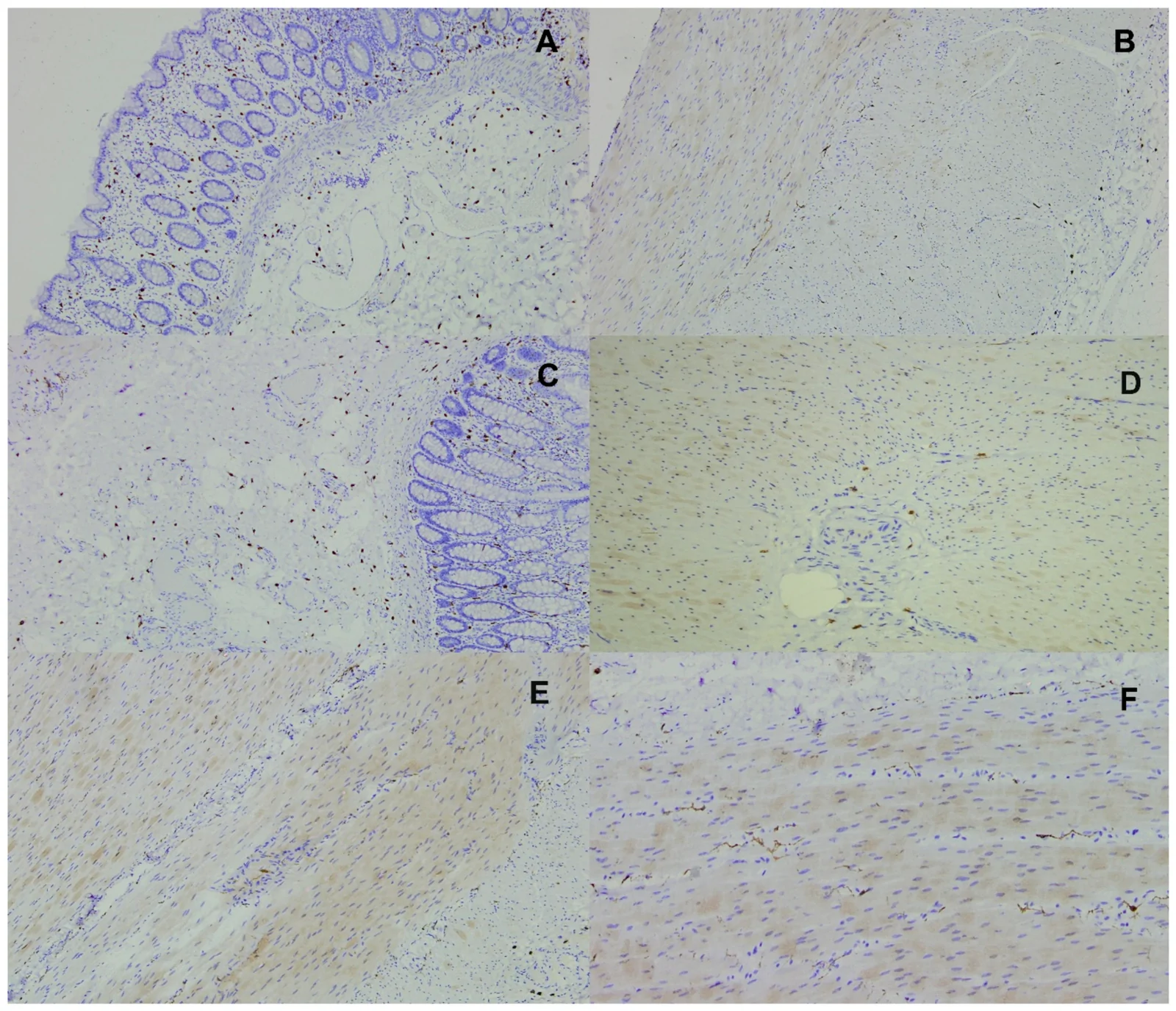
(**A**,**B**) Cajal cells in the submucosa and myenteric plexus in the normal bowel and resection lines (CD117, c-kit, 2.5× and 5×); (**C**–**F**) Cajal cells in the submucosa ((**C**), CD117, 5×), myenteric plexus ((**D**,**E**), CD117, 5× and 10×) and the intramuscular plane in the dilated part of the specimen ((**F**), CD 117, 10×).

The histopathological examination was performed using Formalin-Fixed Paraffin-Embedded (FFPE) tissue, cut at 3 µm. Immunohistochemistry was performed on an autostainer Leica Bond (Nussloch, Germany) with ready-to-use reagents—CD117 clone EP10 and S100 clone EP32. Calretinin, D2-40 and SOX10 were also used. The dilated part revealed a fibrotic submucosa with hyperplastic vessels and nonspecific inflammatory changes, hypertrophy of the muscle layers and nerve plexuses, and giant ganglia in both submucosal and myenteric plexuses (Figure 4). Of note, the same picture was observed in both the distal narrowed part and the resection lines. There was no obvious abnormality of the Cajal cells (morphology and counts) in both parts of the specimen (Figure 5). In the affected, dilated area of the colon, there were between 12 and 18 cells per field in three high-power fields (20× magnification), whereas in the resection margins, there were between 9 and 13 cells per field in three high-power fields (20× magnification).

A final diagnosis of idiopathic megacolon was made based on the lack of an organic cause, colonoscopy, intraoperative findings, and histopathology.

## 3. Discussion

IM is a rare disease. Spanning the period from 1924 to 2017 and using strict selection criteria, the excellent systematic review by Cuda et al. found only 23 papers reporting on 523 patients in the English-language literature [6]. The condition primarily affects healthy individuals, with a mean age of 52 years and a slight female preponderance.

Even today, the IM remains a diagnosis of exclusion [6]. Of particular importance for the proper diagnosis and management of IM is to exclude the organic causes—distal obstruction, pseudo-obstruction (Ogilvie’s syndrome), infections (toxic megacolon), IBD, Hirschsprung’s disease, Chagas disease, Duchenne’s muscular dystrophy, endocrine disorders, mental illness, neurogenic conditions and drugs [2,3].

The imaging diagnostic is based on a sigmoid diameter greater than 10 cm on X-ray or barium enema [6]. It should be noted that the size of the different parts of the colon differs. Aleshin et al. reported a maximal diameter of 9 cm for the cecum and ascending colon, 8.5 cm for the transverse colon, 6 cm for the descending and sigmoid colon and 8.5 cm for the rectum [4]. The X-ray is the routine initial step and may demonstrate a dilated sigmoid. Colonoscopy is often incomplete due to a dilated, elongated sigmoid, although it remains helpful for excluding organic disease, such as Hirschsprung disease. The sigmoid is the most frequent localisation, but IM may also affect the proximal colon. In such cases, contrast CT is the method of choice for evaluating the remaining bowel.

There is no specific conservative treatment. The common arsenal consists of bulking agents, osmotic or stimulant laxatives, enemas, and prokinetics [7]. Surgery is indicated when the conservative treatment has failed or is poorly tolerated or when complications occur. Depending on the affected segment, it could be sigmoidectomy (segmental resection), subtotal colectomy, anterior rectal resection, proctectomy, Duhamel procedure, or restorative coloproctectomy. Gladman et al. reviewed 27 papers published in 1961–2001 and concluded that “recommendations based on firm evidence cannot be given” [8]. Most studies were small series; half of them failed to demonstrate dilated bowel or to exclude an underlying organic cause. The success, defined as “relief of the symptoms and obstipation not requiring laxatives,” varied widely within and between treatment groups. The subtotal colectomy (n = 142 patients) was associated with 71% success, but with significant morbidity (24%), further surgery in 15% and a high rate of recurrent bowel obstruction requiring surgery (15%). The segmental resection (n = 61 patients) had a lower success rate (48%), with further surgery required in 24% of cases. Still, the authors clearly stated that the analysis of this subgroup is confusing and unreliable due to the “tendency to summarize the outcome of segmental with subtotal resection” [8]. Nevertheless, even with caution, they recommend subtotal instead of segmental resection due to the high rate of postoperative constipation in the latter. In contrast, the two presented cases had an excellent quality of life, without constipation or other complaints. Among the other procedures, Duhamel was the most frequent, with 87% success, 29% morbidity and further surgery in 7% [8]. However, the authors do not recommend this procedure due to an unacceptably high morbidity and mortality. To date, there is no reliable tool for functional colon investigation that can aid in choosing a method [8].

According to the London classification of gastrointestinal neuromuscular pathology (GINMP), there are three main groups of bowel neuromuscular disorders: neuropathies, myopathies, and abnormalities of intestinal Cajal cells (enteric mesenchymopathy) [5]. The most common neuropathies include absent neurons, decreased or increased neuron numbers, and degenerative neuropathy. An essential stipulation of the authors is that morphological abnormalities “provide weak evidence of the pathogenetic mechanism and may or may not be causally related to the clinical entities”.

Histopathology of IM in the published series is highly variable, and its specificity remains uncertain. It could be associated with the following histopathological phenotypes: hypoganglionosis, degenerative neuropathy, intestinal neuronal dysplasia type B, abnormal neurochemical coding, degenerative leiomyopathy, muscularis mucosae hypertrophy, filament protein abnormalities, and atrophic desmosis [5]. The main question is whether the neuronal changes are a cause or a consequence of megacolon and longstanding constipation. Cuda et al. found 11 papers reporting histological findings ranging from normal histology to decreased interstitial cells of Cajal (ICC), diminished ganglia, and neuronal densities [6]. In a detailed analysis to assess the leading causes of IM, Ohkubo et al. found hypoganglionosis in 61%, myopathy in 36%, and mesenchymopathy in 32% of cases [9]. They first demonstrated similar abnormalities in the dilated and non-dilated colon (83–100% concordance rate) and speculated that “histopathologic abnormalities underlie IMC patients before clinical manifestation and consequently cause colonic dilatation” [9]. It is unclear why segmental IM affects a particular site more frequently, especially the sigmoid. The presumed cause is that the sigmoid is not attached to the retroperitoneum and has increased intraluminal pressure due to the sphincter of O’Beirne [10]. However, this is only speculation, as in one of the most extensive series by Ohkubo et al., the ascending colon was affected in 45% of cases (14/31). In contrast, descending and sigmoid megacolon were observed in 29% and 26%, respectively [9].

A possible cause of IM could be Intestinal Neuronal Dysplasia (IND). In 1970, Nezelof et al. described hyperplasia of the myenteric plexus, but Meier-Ruge later coined the term “IND” in 1971 [11]. Nowadays, it is referred to as a pathology of the submucosal plexus, characterised by the following quantitative diagnostic criteria: the presence of 15–20% giant ganglia (>8 neurons per ganglion) in 30 sections (four to seven, usually five ganglia) in patients older than one year [12,13]. Type B is observed in approximately 95% of cases and primarily affects adults. IND must be clearly distinguished from ganglioneuromatosis (MEN2B, ganglia with >20 nerve cells). For diagnosis, Meier-Ruge et al. suggest using a ticker (15 µm) cryostat sections. The authors warn of the risk of false diagnosis in tangential biopsy, where the disciform appearance of ganglia can lead to a false-positive result.

We performed a literature search in PubMed from 1975 (when Meier-Ruge established the criteria for IND) to 2025, using the term “intestinal neuronal dysplasia in adults”. Nineteen out of 73 papers reported cases with IND. The full text of two of them could not be retrieved. Of the remaining 17 papers, six were excluded because they reported a paediatric series or only biopsy samples, leaving 11 that reported IND type B (Table 1).

Of the 112 patients, 68 were reported to have IND. In our opinion, the histological report in three papers (32 patients) does not meet the criteria for IND [15,23,24]. If we exclude them, 36 patients remain, of whom four had IND and megacolon (11%) [18,19,20,22]. The vast majority of the patients (n = 30) had chronic constipation—24 patients in the series of Stoss et al. and six with slow-transit constipation in the series of Han et al. [14,17]. In one case, the authors described a palpable mass in the right lower quadrant [16]. Analysing the literature, one important consideration appears. The authors did not describe in detail the pathological methods (the thickness of the sections and the percent of giant ganglia). For example, Vougas et al. reported “enlarged myenteric and submucosal neurons as well as an increased number of giant cells” and “a small number of isolated ganglion cells and giant submucosal ganglia (containing 7–15 ganglion cells) in the submucosa” [20]. A similar issue can be found in the other three cases [18,19,22]. In 2022, Giambusso et al. reported another case of perforated megacolon. Although the pathological report was not well described, they did not report IND [2]. Last but not least, a nationwide survey in Japan clearly demonstrated the rarity of IND type B [25]. The authors reported only 18 cases; however, only four met the IND criteria (1%).

On the other hand, IND remains a matter of debate. A large body of evidence suggests that IND is not a distinct entity and that the morphological changes may be secondary. Back in 1993, Berry questioned the existence of IND [26]. In 1998, Lumb and Moore, reviewing the literature, clearly demonstrated several inconsistencies—the same number of giant ganglia in constipated and non-constipated adults, lack of correlation between histological criteria for IND and clinical outcome, unclear function of submucous plexus (rather secretomotor)—and suggested “to go back to the drawing board” [27]. Moreover, they clearly demonstrated that giant ganglia were present in 78% of the subjects with a normal bowel, with a rate similar to that in IND (10%), even in 3 µm sections stained with haematoxylin and eosin [28]. In the 2003 review, Kapur again highlighted the lack of clinical correlation and concluded that “the presence of IND-like changes is insufficient reason for intestinal resection” [29]. In our opinion, the critical analysis of the literature does not support IND as a possible cause of IM.

In the presented cases, there was hypertrophy of both plexuses and a presence of giant ganglia, but with a similar number in both dilated and normal bowels. Moreover, they were also present in the submucosal and myenteric plexuses. Based on this finding, although 3 µm sections were used, we do not support the diagnosis of IND. Moreover, a similar finding was reported by Lumb and Moore [28].

The ICCs may play a role in the pathophysiology of IM, as their role in bowel motility is well established. ICCs originate from the mesoderm and serve as a pacemaker of bowel motility, providing continuous electrical slow waves. They are located in the submucosal and myenteric plexuses, as well as intramuscularly in both muscles and subserosa [10]. The submuscular ICCs are the dominant cells generating the so-called “slow wave” with rhythmic depolarisation at three cycles/min, named “the signature of ICC pacemaker activity” by Huizinga et al. [10]. This activity causes propagating but not propulsive activity. The transit (propulsion) is secured by the high-amplitude propagating pressure wave, which is driven by the extrinsic autonomic nervous system. In this scenario, the myenteric ICCs are not primary, but stimulus-dependent pacemakers. Simultaneous pressure waves are generated by the parasympathetic nervous system and propagate throughout the colon via the myenteric ICCs. The submucosal and myenteric ICCs also generate the so-called cyclic motor pattern in the sigmoid and rectum, which is retrograde and thus helps continence.

Impaired ICC function may lead to slow transit and subsequent dilatation [6]. This electrical activity may be lost due to various factors, including infections, loss of c-kit expression, and autoimmune processes [10]. Their role in diabetic gastroparesis is well known [27]. Lyford et al. demonstrated significantly lower ICC volume in all colonic parts in slow-transit constipation [30]. According to the review by Hiuzinga et al., several other authors have reported similar findings in 2021 [10]. The review of Knowles et al. demonstrated that the reduction in ICC is the most consistent histological finding in slow-transit constipation [31].

A similar finding has also been demonstrated in IM. In four patients, Adachi et al. found decreased c-kit expression but preserved CD34+ expression in ICC, which was associated with slow-transit constipation [32]. Ohkubo et al. found a reduction in more than 50% or total loss of ICC in one-third of the dilated colon in their series, but with a similar rate in the normal colon [9]. Nevertheless, according to the 2009 International Working Group, the aetiological role of ICC in gastrointestinal neuromuscular diseases remains unclear [33].

Another essential issue is the lack of standardisation of methods for detecting the ICC. In contrast to haematoxylin–eosin, immunohistochemistry found additional neuronal abnormalities in 83% of the cases [9]. Lyford et al., however, state that paraffin embedding decreases c-kit staining, so they froze the tissues immediately after procurement at −70 degrees until processing [31]. Using immunofluorescence on frozen tissues, Garrity et al. found 42% more ICCs in the circular muscle layer, 38% in the longitudinal layer, and 42% in the myenteric plexus [34].

Another issue is the quantitative assessment of ICCs. A loss of more than 50% of ICCs seems to be clinically relevant [5]. But what is the “normal” range? Gomez-Pinilla et al. found a 5-fold higher density in the myenteric plexus than in the circular muscle, whereas others found more ICCs in the circular muscle [33,34]. On fresh-frozen sections, Garrity et al. demonstrate a mean of 15 ICC/hpf in the circular muscle, 8.7 in the longitudinal, and 10.2 in the myenteric plexus, whereas Gomez-Pinilla et al. found 5.5 in the circular muscle and 7.9 in the myenteric plexus [33,34]. Moreover, it is crucial to minimise the ischemic time between specimen procurement and fixation. The issue is further complicated because the ICC number appears age-dependent, with 13% loss per decade of life, a finding that is not affected by sex or colonic region [34]. Last but not least, the loss of c-kit does not always indicate a loss of ICC.

We could not find myopathy or ICC abnormalities (normal morphology and count according to the definition of London classification, 2009) [5]. The changes were presented in both dilated and non-dilated bowels, similar to those described by Ohkubo et al. [9].

Despite familial clustering in some cases, to date, there is no conclusive evidence of an association between IM and a particular gene [1]. Due to the high heterogeneity of colonic motility disorders and the lack of a consistent histological pattern in IM, we hypothesise that IM has a multifactorial aetiology.

Limitations of the present study are related to the description of only two cases, which precludes the generalisation of the findings. The specimens were cut at 3 µm instead of 15 µm, so IND could not be excluded. Yet, we consider this diagnosis speculative.

## 4. Conclusions

We were unable to identify a specific cause of IM in the presented cases. The histopathological finding was not compatible with IND, Hirschsprung disease, or other neuromuscular pathology. The literature review demonstrated that only 11% of INDs were associated with IM. Nevertheless, the diagnosis of IND should be considered with caution due to the incomplete pathological reports. The critical analysis of the literature does not support IND as a possible cause of IM.

Due to the high heterogeneity of colonic motility disorders and the lack of a consistent histological pattern in IM, we hypothesise that IM has a multifactorial aetiology. As a literature-based hypothesis, it can be speculated that Cajal’s cell abnormalities may contribute to the pathophysiology of IM.

The work complied with the Declaration of Helsinki; patient privacy was protected, and written informed consent was obtained for the publication of the clinical information and images.

Ethical review and approval of this study were not required (or waived) by the Ethics Committee of the Military Medical Academy because case reports are not considered research. The patient’s information has been de-identified. Both patients consented to their cases being published.

## Figures and Tables

**Table 1:** Characteristics of the included studies reporting IND type B.

Author	n	Case Description	MC	Procedure	Microscopic Examination
Stoss, 1994 [14]	41	chronic constipation	no	21/36 operated: sigmoidectomy (2), internal sphincterotomy (12), subtotal colectomy (7)	IND in 24 (57%)
Barnes, 1986 * [15]	30	pain, distension, infrequent bowel actions (19), diarrhoea (7); previous sigmoid colectomy in 6, left hemicolectomy in 1, colectomy with cecorectal anastomosis in 4	yes	surgery in 22 patients: colectomy with cecorectal anastomosis (10) colectomy with ileorectal anastomosis (8)	“disorder of the myenteric plexus”
Wu, 1997 [16]	1	46-year-old man palpable mass in the right lower quadrant	no	right colectomy	IND
Han, 2012 [17]	33	chronic constipation with colonic pseudo-obstruction (CPO)	no	total colectomy (32), transversostomy (1)	CPO group: hypoganglionosis (HG) or agangliosis (AG) in 23 patients, and HG combined with visceral neuropathy or myopathy in 10 patients; slow-transit consipation group: IND type B (n = 6) and visceral myopathy (n = 3)
Banares, 2014 [18]	1	33-year-old man sub-occlusive episodes since childhood, right colectomy with ileostomy and left colostomy	yes	ileal resection with side-to-side ileocolonic anastomosis, re-resection with re-anastomosis	IND and a decrease in interstitial cells of Cajal
López Sanclemente, 2014 [19]	1	47-year-old man severe abdominal distension and respiratory failure	yes	colectomy with ileorectal anastomosis	IND
Vougas, 2014 [20]	1	71-year-old man abdominal pain and constipation	yes	subtotal colectomy—Hartman’s procedure	IND
Hokama, 2019 [21]	1	27-year-old man chronic abdominal distension	yes	colectomy with ileorectal anastomosis	IND
Masuda, 2017 [22]	1	36-year-old man severe constipation for 20 years	yes	SILS colectomy with ileorectal anastomosis	IND
Ren, 2022 * [23]	1	psoas abscess in a 31-year-old man	no	ileostomy	“hyperplastic nerve fibers in the submucosa and the muscular layer”
Khan, 2023 * [24]	1	31-year-old woman distension, pain, constipation	yes	total colectomy with ileostomy	“ganglionated plexuses with very subtle alterations in morphology, size and number of ganglia. A few crowded miniatured ganglia were seen along with giant ganglia.”

* Unclear criteria/report, which does not meet the criteria for IND type B.

## Data Availability

The original data presented in the study are included in the article; further inquiries can be directed to the corresponding author.
